# Antagonist affinity measurements at the Gi-coupled human histamine H3 receptor expressed in CHO cells

**DOI:** 10.1186/1471-2210-8-9

**Published:** 2008-06-06

**Authors:** Jillian G Baker

**Affiliations:** 1 Institute of Cell Signalling, Medical School, University of Nottingham, Queen's Medical Centre, Nottingham, NG7 2UH, UK

## Abstract

**Background:**

The H3 histamine receptor is a Gi-coupled GPCR that has been proven to exist in different agonist-induced states, including that defined by the protean agonist proxyfan. Several GPCRs are now known to exist in different states. For some of these, antagonist affinity measurement remain constant regardless of the state of the receptor, for others e.g. the beta-adrenoceptors, the antagonist affinity measurements vary considerably depending on which agonist-dependent state is being identified. The purpose of this study was to examine the antagonist affinity measurements at the Gi-coupling human H3 receptor, paying particular attention to measurements made in the presence of full agonists, partial agonists and the proxyfan protean agonist-induced state of the receptor.

**Results:**

CHO cells stably expressing the human histamine H3 receptor and a CRE-SPAP reporter were used. Measurements of CRE-gene transcription and ^3^H-cAMP accumulation were made. A range of ligands of different agonist efficacies were determined, including some partial agonists e.g. VUF 5681. Unlike other Gi-coupled receptors, no Gs-coupled state of the receptor was detected with these ligands. Antagonist affinity measurements were constant, whether the measurements were made in the presence of a full agonist, a partial agonist or the protean agonist proxyfan.

**Conclusion:**

In contrast to all three subtypes of the beta-adrenoceptors, but in keeping with the traditional pharmacological dogma, antagonist affinity measurements remained constant at the human H3 receptor, including the medium-efficacy proxyfan-induced state of the receptor and the VUF5681-induced state of the receptor.

## Background

Antagonist affinity measurements have always played a key role in pharmacology [1-3]. They have been vital in the initial determination of different receptor sub-types and have been a major tool in determining which receptor subtypes are present within a given tissue (e.g. [1,4,5]. But these definitions have been made on the assumption that antagonist affinity measurements remain stable for a given ligand-receptor interaction, regardless of the competing agonist or the assay used to determine it.

Recently it has been shown that antagonist affinity measurements do not remain constant at the β-adrenoceptors [6-8]. Antagonist affinity measurements at the human β1-adrenoceptor may vary up to 3000 fold within the same experimental set up purely depending on which ligand is the competing agonist (e.g. [9-13]). Initially this led to the suggestion of the existence of a β4-adrenoceptor, however many studies since, including those using knockout animals [14,15], have shown that the different antagonist affinities determined are from one receptor, the β1-adrenoceptor, but that this receptor exists in at least two different agonist states [6-8].

Several GPCRs have now been shown to exist in more that one agonist state or conformation.

For some receptors, this results in signalling via more than one class of G-protein (e.g. [16-18]. For other GPCRs, the different agonist-induced states of the receptor have different abilities to bind antagonists, e.g. β1 and β3-adrenoceptors [12,19]. The human β2-adrenoceptor also appears to exist in at least two-agonist induced states, for which antagonist affinity measurements vary in CRE-gene transcription assays. However the reason for this remains unclear but may result from a time-dependent efficacy-related process [20]. Some GPCRs appear to differentially activate different signalling cascades and must also exist in different states [21-24]. Finally studies to date with other GCPRs do not detect the existence of any other state (at least with the ligands currently available) and are therefore considered to exist in a single agonist conformation with a constant antagonist affinity [25].

The histamine H3 receptor is a constitutively active GPCR that is involved in neurotransmitter release within the CNS [26] and references therein. The histamine receptor subtypes H1 and H2 were first characterised in the 1960's and 1970's [2,5]. Then in the 1970's and early 1980's, Arrang, Schwartz and colleagues observed that not all histamine actions in rat neurones were inhibited by H1 and H2 antagonists in a manner that fitted the profile for either histamine receptor subtype and therefore proposed a third histamine receptor subtype [26,27]. The existence of the histamine H3 receptor was finally confirmed by the demonstration that R-α-methylhistamine was more potent than S-α-methylhistamine in reducing histamine release from rat brain slices and the identification of thioperamide as a selective H3 antagonist [28]. The H3 receptor was cloned in 1999 [29] and many selective and very high potency H3 agonists and antagonists have been identified [30]. The H3 receptor couples to heterotrimeric Gi/o-proteins, activation of which causes a decrease in cAMP production and PKA activation [31]. Its other known actions (including activation of phospholipase A2, Akt/GSK-3β axis and MAPK pathways, inhibition of the Na+/H+ exchanger and modulation of intracellular calcium) are all thought to occur via the various subunits of the heterotrimeric G-protein following Gi/o activation [31].

Recently, several unusual properties for the histamine H3 receptor and its ligands have been demonstrated. Of particularly interest here is that the H3 receptor exists in different agonist-induced states. Proxyfan has been demonstrated to be a protean agonist [32] of the H3 receptor – thus the efficacy observed depends upon the receptor expression level and constitutive activity in the system under study [33]. Proxyfan stabilises a medium-efficacy state of the H3 receptor and so appears as a full or partial agonist in systems with less constitutive activity, a neutral antagonist of higher efficacy ligands in lower receptor expression systems, and an inverse agonist in highly constitutively active system, where it stabilises a receptor conformation of lower efficacy than the constitutively active state [33]. Thus proxyfan has conclusively demonstrated that the histamine H3 receptor exists in more than one agonist state or conformation.

The aim of this study was therefore to examine the antagonist affinity measurements made at the human H3 histamine receptor stably expressed in CHO cells using a CRE-reporter gene system. This study examines, in detail, antagonist affinity measurements made at the different states of the H3 receptor including those induced by partial agonists, and at the medium-efficacy proxyfan induced state as distinct from the full-agonist histamine induced state of the receptor.

## Methods

### Materials

Fetal calf serum was from PAA laboratories (Teddington, Middlesex, UK). ^3^H-adenine and ^14^C-cAMP were from Amersham International (Buckinghamshire, UK). N-α-methylhistamine, R-α-methylhistamine, S-α-methylhistamine, 4-methylhistamine, immepip, imetit, immethridine, impentamine, amthamine, dimaprit, VUF 5681, VUF 8430, HTMT, 2-pyridylethylamine, iodophenpropit, clobenpropit, proxyfan, zolatidine, conessine, thioperamide, dimethindene, triprolidine, clemastine and burimamide were obtained from Tocris Cookson (Avonmounth, Bristol, UK). Pertussin toxin was from Calbiochem (Nottingham, UK). Histamine, cimetidine, ranitidine, 1-methylhistamine, 3-methylhistamine, chlorpheniramine, IBMX and forskolin were from Sigma Chemicals (Poole, Dorset, UK) who also supplied all other reagents.

### Cell Culture

A stable clonal CHO-K1 cell line expressing a CRE-SPAP reporter gene (six CRE upstream of a SPAP reporter) was secondarily transfected with the full length human histamine H3 receptor (DNA from DNA from UMR cDNA Resource Centre) using Lipofectamine and OPTIMEM as per manufacturer's instructions. The transfected cells were selected for neomycin resistance (1 mg/ml; for H3 receptor) and hygromycin resistance (200 μg/ml; for CRE-SPAP reporter gene) for 3 weeks and passaged twice during in this period. A single clone was then isolated by dilution cloning to give a clonal line (CHO-H3-SPAP cells). The parent cell CRE-SPAP was also used for control experiments (CHO-SPAP cells). Cells were grown in Dulbecco's modified Eagles medium/Nutrient mix F12 (DMEM/F12) containing 10% fetal calf serum and 2 mM L-glutamine at 37°C in a humidified 5% CO_2_: 95% air atmosphere.

### ^3^H-R-α-methylhistamine whole cell binding

Cells were grown to confluence in white-sided 96-well view plates. The media was then removed and replaced with 100 μl ice cold PBS (4°C) per well (total binding) or 100 μl ice cold PBS (4°C) containing 1 μM iodophenpropit (non-specific binding). 100 μl ^3^H-R-α-methylhistamine was then immediately added to the wells to give concentrations in the range of 0.024–224 nM. Cells were then maintained a 4°C for 5 hours. All PBS and drugs were removed and the cells washed twice by the addition and removal of 2 × 200 μl 4°C PBS. A white bottom and clear sealant top was added to the wells, the plates left overnight in the dark at room temperature and the plates counted on a Topcount (Packard) at 21°C 2 minute count per well.

### CRE-SPAP gene transcription

Cells were grown to confluence in 96-well tissue culture plates. The cells were then serum starved by removing the media and replacing it with 100 μl serum free media (DMEM/F12 containing 2 mM L-glutamine). The cells were then incubated for a further 24 hours (humidified 5% CO_2_: 95% air atmosphere at 37°C). Where used, pertussis toxin, at a final concentration of 100 ng/ml was added to the serum-free media and thus incubated with the cells for 24 hours. On the day of experimentation, the serum-free media was removed and replaced with 100 μl serum-free media or 100 μl serum-free media containing an antagonist at the final required concentration and the cells incubated for 1 hour. Agonist in 10 μl (diluted in serum free media) was then added to each well and the cells incubated for 10 minutes. Forskolin was then added to all but the basal wells to give a final well concentration of 4 μM and the plates incubated for 5 hours. After 5 hours, the media and all drugs were removed. 40 μl serum-free media was added to each well and the cells incubated for a further 1 hour (37°C, 5% CO_2_: 95% air atmosphere). The plates were then incubated at 65°C for 30 minutes to destroy any endogenous phosphatases. After cooling to 37°C, 100 μl 5 mM pNPP in diethanolamine buffer was added to each well and the plates incubated at 37°C in a normal atmosphere until the yellow colour developed. The plates were then read on a Dynatech MRX plate reader at 405 nm.

### ^3^H-cAMP accumulation

Cells were grown to confluence in 24-well plates. The media was removed and the cells pre-labelled with ^3^H-adenine by incubation with 2 μCi/ml ^3^H-adenine in serum-free media (0.5 ml per well) for 3 hours. The ^3^H-adenine was then removed and the cells washed by the addition then removal of 1 ml serum-free media. 1 ml serum-free media containing 100 μM IBMX with or without the final required concentration of antagonist was then added to each well and the cells incubated for 30 minutes. Agonist (in 10 μl serum-free media) was added to each well and the plates incubated for 10 minutes. Forskolin (final concentration of 10 μM) was then added to all but the basal wells and the plates incubated for 30 minutes. The reaction was terminated by the addition of 50 μl concentrated HCl per well and the plates were then frozen. Later, the plates were thawed and ^3^H-cAMP separated from other ^3^H-nucleotides by sequential Dowex and alumina column chromatography, as previously described [34].

When the intrinsic efficacy of the antagonist ligands was examined, the antagonists were incubated for 5 hours at 37°C in order to maximise the chance of detecting any changes in ^3^H-cAMP accumulation.

### Data Analysis

#### Binding studies

To determine the expression level in the CHO-H3-SPAP cells, the saturation curves for specific ^3^H-R-α-methylhistamine binding were fitted to the following equation using GraphPad Prism 2:

$$\text{Specific binding}=\frac{{\text{B}}_{\max}({[}^{3}\text{H-R}\alpha \text{mh}])}{\left({[}^{3}\text{H-R}\alpha \text{mh}]+{\text{K}}_{\text{D}}\right)}$$

However, as the specific binding curves appeared to contain a linear component, the data were also fitted to the following expression:

$$\text{Specific binding}=\frac{{\text{B}}_{\max}({[}^{3}\text{H-R}\alpha \text{mh}])}{\left({[}^{3}\text{H-R}\alpha \text{mh}]+{\text{K}}_{\text{D}}\right)}+\text{M}\times ({[}^{3}\text{H-R}\alpha \text{mh}])$$

B_max _is the maximum specific binding, K_D _is the dissociation constant of ^3^H-R-α-methylhistamine, [^3^H-Rαmh] is the concentration of ^3^H-R-α-methylhistamine and M is the slop of the linear component of binding.

#### Functional data

Sigmoidal concentration-response curves were fitted to the data using Graphpad Prism 2 and the following equation:

$$\text{Response}=\frac{\text{Emax}\times [\text{A}]}{{\text{IC}}_{50}+[\text{A}]}$$

where Emax is the maximal response, [A] is the agonist concentration and IC_50 _is the concentration of agonist that produces 50% of the maximal response.

Antagonist K_D _values were then calculated from the shift of the agonist concentration responses in the presence of a fixed concentration of antagonist using the following equation:

$$\text{DR}=1+\frac{\left[\text{B}\right]}{{\text{K}}_{\text{D}}}$$

where DR (dose ratio) is the ratio of the agonist concentration required to stimulate an identical response in the presence and absence of a fixed concentration of antagonist [B].

In experiments where three different fixed concentrations of the same antagonist were used, Schild plots were constructed using the following equation:

Log (DR-1) = log [B] - log (K_D_)

These points were then fitted to a straight line. A slope of 1 then indicates competitive antagonism [1].

When VUF 5681 was used as an antagonist (e.g. Figure 4), the partial agonist nature of this ligand was seen. Partial agonist dissociation constants were therefore estimated according to the method of Stephenson [35] using the following equation:

$$\begin{array}{cc} {\text{K}}_{\text{D}}\text{ partial agonist}=\frac{\text{X}.[\text{P}]}{1-\text{X}} & \text{where X}=\frac{\left[{\text{A}}_{2}]-[{\text{A}}_{1}\right]}{\left[{\text{A}}_{3}\right]} \end{array}$$

where [P] in the concentration of the partial agonist VUF 5681, [A_1_] in the concentration of the agonist at the point where the fixed partial agonist causes the same response, [A_2_] in the concentration of agonist causing a given response above that achieved by the partial agonist and [A_3_] the concentration of the agonist, in the presence of the partial agonist, causing the same stimulation as [A_2_].

In Figure 6a, the concentration-response curve is best fitted to a 2-component response, the following equation was used:

$$\%\text{ maximal stimulation}=\frac{[\text{A}].\text{N}}{\left([\text{A}]+\text{IC}1_{50}\right)}+\frac{\left[\text{A}].(100-\text{N}\right)}{\left([\text{A}]+\text{IC}2_{50}\right)}$$

where N is the percentage of site 1, [A] is the concentration of agonist and IC1_50 _and IC2_50 _are the respective IC_50 _values for the two agonist sites.

All data are presented as mean ± s.e.m. of triplicate determinations where n is the number of separate experiments.

## Results

### Determination of CHO-H3-SPAP cell line H3-receptor expression level

From the saturation binding studies, the K_D _value for ^3^H-R-α-methylhistamine was 4.10 ± 0.62 nM (n = 7). The receptor expression level of the human H3 receptor as determined by ^3^H-R-α-methylhistamine was 72.6 ± 8.0 fmol/mg protein (n = 7).

### Identification of H3 agonists

**Figure 1:**
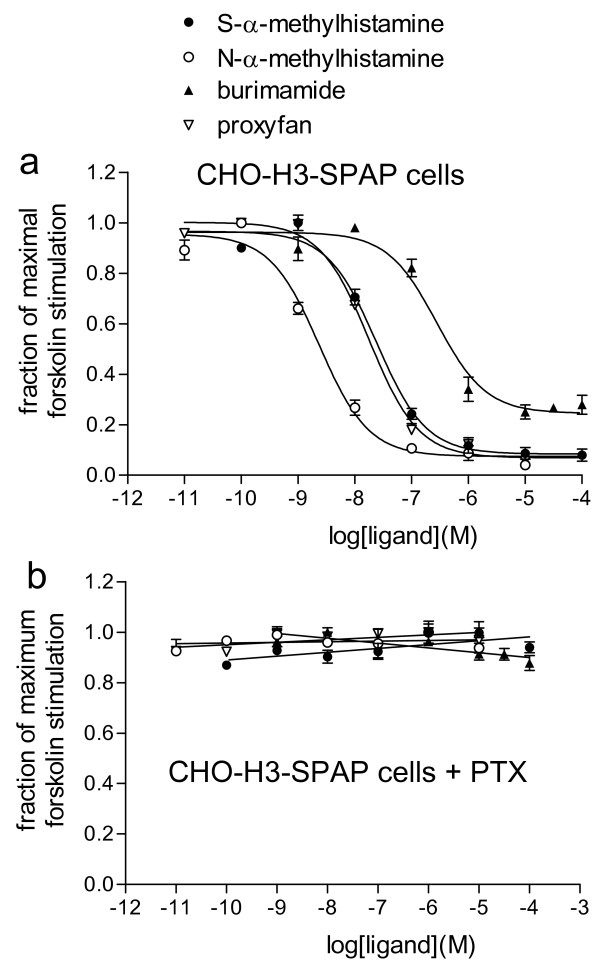
**a) and b) CRE-SPAP production in forskolin-stimulated CHO-H3-SPAP cells in response to S-α-methylhistamine, N-α-methylhistamine and burimamide in the absence a) and following pre-incubation with PTX b).** The figures are normalised to maximum forskolin stimulation where basal = 0 and maximum forskolin stimulation = 1. Data points are mean ± s.e.m. of triplicate values from a single experiment and are representative of a) 6 and b) 3 separate experiments.

Histamine agonists were first screened for inhibition of forskolin-stimulated CRE-SPAP production in CHO-H3-SPAP cells. In addition to the many ligands traditionally considered to be H3 agonists, several others were found to have partial agonist actions – e.g. burimamide and impentamine in keeping with previous studies (Figure 1a, Table 1). VUF 5681 was found to also have agonist properties. In addition the H2 agonists amthamine and dimaprit stimulated agonist responses (pIC_50 _= 4.87 ± 0.08, n = 3; pIC_50 _= 5.59 ± 0.18, n = 6, respectively) as well as HTMT (an H2-agonist [36] pIC_50 _= 5.21 ± 0.18, n = 4). 1-methylhistamine (pIC_50 _= 4.70 ± 0.07, n = 4), 3-methylhistamine (pIC_50 _= 4.87 ± 0.05, n = 5) and 4-methylhistamine (pIC_50 _= 4.94 ± 0.52, n = 4) also stimulated low potency responses. The H1 agonist 2-pyridylethylamine [37] stimulated a response, however the IC_50 _for this was greater than 100 μM.

**Table 1:** pIC_50 _values for H3 agonism

	^3^H-cAMP accumulation		CRE-SPAP	
agonist	pIC_50_	n	pIC_50_	n

histamine	8.55 ± 0.05	12	7.89 ± 0.04	19
N-α-methylhistamine	9.45 ± 0.05	5	8.89 ± 0.04	17
R-α-methylhistamine	9.54 ± 0.06	4	8.94 ± 0.04	16
S-α-methylhistamine	8.31 ± 0.01	5	7.82 ± 0.06	17
imetit	9.91 ± 0.05	5	9.36 ± 0.04	16
immepip	10.30 ± 0.14	5	9.65 ± 0.03	15
immethridine	9.76 ± 0.08	5	9.41 ± 0.04	10
proxyfan	8.31 ± 0.11	5	7.79 ± 0.06	10
VUF 8430	6.38 ± 0.07	6	5.66 ± 0.08	8
Impentamine*	8.43 ± 0.08	5	Site 1 7.59 ± 0.05	8
			Site 2 5.40 ± 0.05	8
burimamide	6.65 ± 0.10	5	6.37 ± 0.11	6
VUF 5681	8.41 ± 0.10	5	7.60 ± 0.13	5

pIC_50 _values for ^3^H-cAMP accumulation and CRE-SPAP production for the ligands found to be agonists at the histamine H3 receptor in CHO-H3-SPAP cells. Values represent mean ± s.e.mean of n separate experiments. * the CRE-SPAP response to impentamine appeared to consist of 2 components (Figure 6a). Of this 50.2 ± 2.0% was occurring via site 1

### Investigation of Gi and Gs coupling

All agonist responses were also examined following 24 hours pre-incubation with pertussis toxin (PTX). Agonist responses to all of the ligands mentioned above and listed in Table 1 were abolished by PTX (Figure 1b). None of the antagonist ligands used in this study stimulated any response either in the absence or following 24 hours pre-incubation with PTX.

Finally all ligands used in the study (agonists and antagonists) were examined in the absence of forskolin (with and without PTX pre-incubation) in order to look for any Gs-stimulatory responses than might otherwise have been masked by the presence of 4 μM forskolin. No responses were seen to any of the ligands (with the exception of impentamine, see below).

### Determination of antagonist affinity

**Figure 2:**
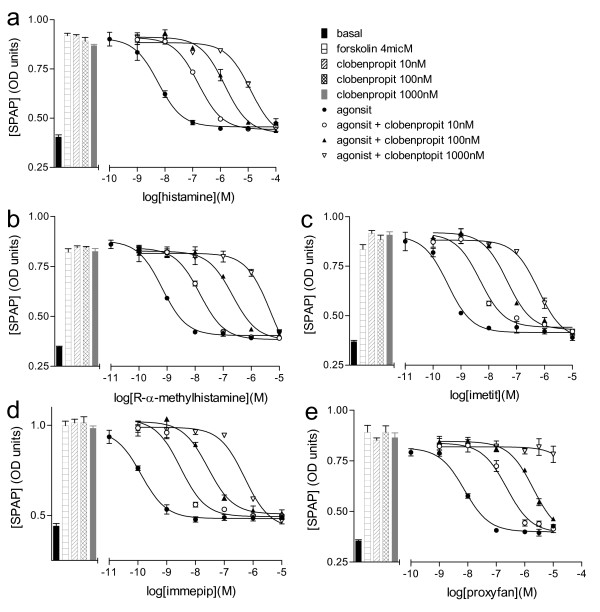
**CRE-SPAP production in forskolin-stimulated CHO-H3-SPAP cells in response to a) histamine, b) R-α-methylhistamine, c) imetit, d) immepip and e) proxyfan in the absence and presence of 10 nM, 100 nM or 1000 nM clobenpropit.** Bars show basal CRE-SPAP production and that in response to 4 μM forskolin alone, and 10 nM, 100 nM or 1000 nM clobenpropit in the presence of 4 μM forskolin. Data points are mean ± s.e.m. of triplicate values from a single experiment and are representative of a) 3, b) 4, c) 4, d) 4 and e) 4 separate experiments.

**Figure 3:**
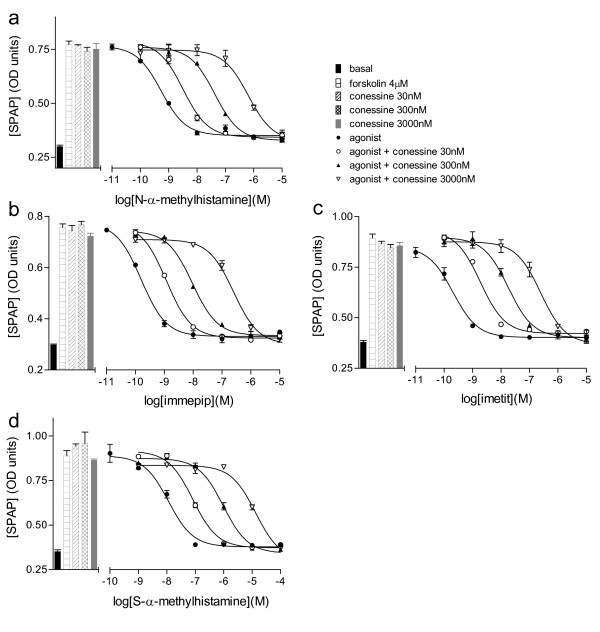
**CRE-SPAP production in forskolin-stimulated CHO-H3-SPAP cells in response to a) N-α-methylhistamine, b) immepip c) imetit and d) S-α-methylhistamine in the absence and presence of 30 nM, 300 nM or 3000 nM conessine.** Bars show basal CRE-SPAP production and that in response to 4 μM forskolin alone, and 30 nM, 300 nM or 3000 nM conessine in the presence of 4 μM forskolin. Data points are mean ± s.e.m. of triplicate values from a single experiment and are representative of 4 separate experiments in each case.

**Figure 4:**
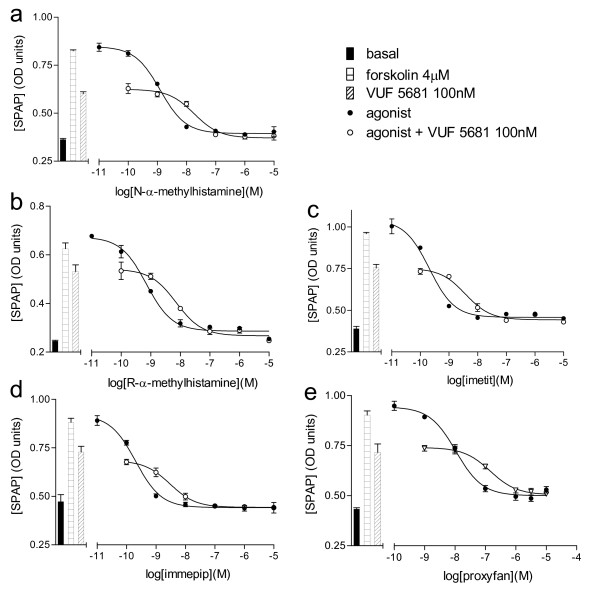
**CRE-SPAP production in forskolin-stimulated CHO-H3-SPAP cells in response to a) N-α-methylhistamine, b) R-α-methylhistamine, c) imetit, d) immepip and e) proxyfan in the absence and presence of 100 nM VUF 5681.** Bars show basal CRE-SPAP production and that in response to 4 μM forskolin alone and 100 nM VUF 5681 in the presence of 4 μM forskolin. Data points are mean ± s.e.m. of triplicate values from a single experiment and are representative of a) 4, b) 4, c) 4, d) 3 and e) 3 separate experiments.

Measurements of antagonist affinity were them made in the presence of agonists of different efficacies, including full agonists, partial agonists and proxyfan (Figures 2 and 3; Table 2). Where possible, the response to each agonist was assessed in the presence of at three different concentrations of antagonists thus allowing a Schild plot to be constructed (Table 3). However, for many antagonist ligands, the affinity was relatively poor and hence antagonist affinity was assessed from parallel shifts in the presence of one or two concentrations of antagonist. The ability of the partial agonist VUF 5681 to inhibit the more efficacious agonists was also assessed (Table 3, Figure 4) however, here the affinity of VUF 5681 was calculated by the partial agonist method of Stephenson [35].

**Table 2:** pK_D _values for H3 antagonism

Agonist	pK_D _values											
	Iodophen-propit	n	Cloben-propit	n	zolatidine	n	conessine	n	thioperamide	n	cimetidine	n

histamine	8.65 ± 0.07	19	9.42 ± 0.04	9	6.51 ± 0.07	19	8.29 ± 0.05	15	7.22 ± 0.04	23	5.64 ± 0.11	11
N-α-methylhistamine	8.59 ± 0.10	16	9.42 ± 0.06	15	6.46 ± 0.07	17	8.27 ± 0.04	12	7.37 ± 0.06	18	5.57 ± 0.08	9
R-α-methylhistamine	8.57 ± 0.09	15	9.21 ± 0.08	15	6.52 ± 0.06	14	8.24 ± 0.06	9	7.33 ± 0.04	17	5.62 ± 0.12	8
S-α-methylhistamine	8.72 ± 0.05	14	9.25 ± 0.05	13	6.54 ± 0.07	18	8.28 ± 0.05	12	7.20 ± 0.05	17	5.64 ± 0.06	9
imetit	8.78 ± 0.06	16	9.20 ± 0.05	12	6.62 ± 0.08	17	8.24 ± 0.10	12	7.29 ± 0.06	13	5.64 ± 0.08	10
immepip	8.66 ± 0.07	14	9.36 ± 0.04	12	6.55 ± 0.07	17	8.09 ± 0.07	12	7.22 ± 0.09	15	5.70 ± 0.09	9
immethridine	8.81 ± 0.08	12	9.21 ± 0.06	12	6.66 ± 0.05	12	8.42 ± 0.10	11	7.31 ± 0.06	12	5.72 ± 0.09	4
proxyfan	8.82 ± 0.05	7	9.25 ± 0.08	7	6.78 ± 0.06	9	8.56 ± 0.08	5	7.59 ± 0.03	9	5.61 ± 0.11	6
VUF 8430	8.53 ± 0.08	6	9.11 ± 0.19	4	6.38 ± 0.10	4	8.15 ± 0.08	4	7.15 ± 0.27	4	5.53 ± 0.13	4
impentamine	8.60 ± 0.09	6	9.01 ± 0.10	4	6.52 ± 0.10	4	8.38 ± 0.11	4	7.47 ± 0.10	5	5.70 ± 0.01	5
												
agonist	Log K_D _values											

	Dimethin-dine	n	Chlorpheni-ramine	n	VUF 5681	n	ranitidine	n	triprolidine	n	clemastine	n

histamine	5.63 ± 0.07	7	5.32 ± 0.06	8	7.77 ± 0.08	5	5.05 ± 0.08	9	5.35 ± 0.05	7	5.73 ± 0.08	10
N-α-methylhistamine	5.67 ± 0.17	6	5.51 ± 0.07	3	7.95 ± 0.14	4	5.05 ± 0.08	9	5.34 ± 0.08	6	5.69 ± 0.13	7
R-α-methylhistamine	5.65 ± 0.04	5	5.44 ± 0.04	4	7.60 ± 0.07	4	5.18 ± 0.06	7	5.58 ± 0.05	5	5.79 ± 0.09	7
S-α-methylhistamine	5.67 ± 0.03	4	5.44 ± 0.08	3	7.75 ± 0.04	4	4.95 ± 0.10	7	5.47 ± 0.08	6	5.74 ± 0.11	8
imetit	5.60 ± 0.16	8	5.58 ± 0.08	4	7.85 ± 0.08	4	4.96 ± 0.09	7	5.43 ± 0.09	7	5.69 ± 0.15	6
immepip	5.72 ± 0.16	5	5.59 ± 0.12	4	7.72 ± 0.10	3	5.12 ± 0.06	6	5.61 ± 0.13	4	5.79 ± 0.15	6
immethridine	5.38 ± 0.05	9	5.53 ± 0.05	4	7.79 ± 0.12	4	5.19 ± 0.09	3	5.53 ± 0.13	3	5.50 ± 0.10	3
proxyfan	5.24 ± 0.14	3	5.39 ± 0.11	5	7.80 ± 0.03	3	4.97 ± 0.12	5	5.23 ± 0.07	5	5.60 ± 0.11	6
VUF 8430	5.11 ± 0.09	3	5.13 ± 0.15	4	7.71 ± 0.16	3	4.72 ± 0.01	3	5.26 ± 0.10	3	5.26 ± 0.06	4
impentamine	5.59 ± 0.24	3	5.21 ± 0.22	4	*		5.10 ± 0.09	5	5.65 ± 0.15	4	5.73 ± 0.09	5

pK_D _values for 12 antagonists as determined from measurements of CRE-SPAP production from CHO-H3-cells made in the presence of the 12 different agonists. Values represent mean ± s.e.mean of n separate experiments. The pK_D _values quoted are for antagonism of the first component of the impentamine response.

* it was not possible to determine a pK_D _value for VUF 5681 in the presence of impentamine due to the partial agonist nature of both responses.

**Table 3:** Schild slopes for the H3-antagonists

agonist	iodophenpropit	n	clobenpropit	n	zolatidine	n	conessine	n	thioperamide	n
histamine	1.15 ± 0.04	5	0.98 ± 0.03	3	1.01 ± 0.04	4	1.07 ± 0.03	5	1.01 ± 0.03	4
N-α-methylhistamine	1.12 ± 0.04	5	1.08 ± 0.07	3	1.10 ± 0.12	4	0.98 ± 0.05	4	0.98 ± 0.07	5
R-α-methylhistamine	1.14 ± 0.05	5	1.10 ± 0.06	4	1.04 ± 0.04	3	1.00 ± 0.06	3	1.00 ± 0.05	5
S-α-methylhistamine	1.17 ± 0.04	4	1.09 ± 0.04	3	1.10 ± 0.07	4	1.08 ± 0.04	4	1.01 ± 0.08	6
Imetit	1.09 ± 0.04	5	1.02 ± 0.04	4	1.17 ± 0.05	4	1.18 ± 0.09	4	1.02 ± 0.07	3
immepip	1.13 ± 0.04	4	1.05 ± 0.02	4	1.18 ± 0.08	4	1.09 ± 0.05	4	1.06 ± 0.02	4
immethridine	1.28 ± 0.13	4	1.02 ± 0.04	4	0.96 ± 0.02	4	1.14 ± 0.08	3	1.08 ± 0.04	4
proxyfan	*		*		0.91 ± 0.11	3	*		1.02 ± 0.03	3

Slope of the Schild plot from antagonism of those compounds where 3 different concentrations of antagonists were possible as determined from measurements of CRE-SPAP production from CHO-H3-SPAP cells. Values represent mean ± s.e.mean of n separate experiments.

* Schild plots were not obtained. Schild plots were also not obtained when impentamine and VUF 8430 were agonists

**Figure 5:**
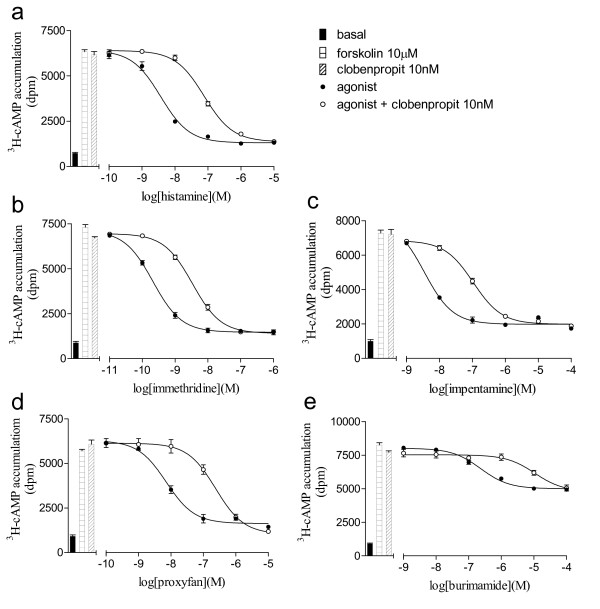
**Forskolin-stimulated ^3^H-cAMP accumulation in CHO-H3-SPAP cells in response to a) histamine, b) immethridine, c) impentamine d) proxyfan and e) burimamide in the absence and presence of 10 nM clobenpropit.** Bars show basal ^3^H-cAMP accumulation, that in response to 10 μM forskolin alone and that in response to 10 nM clobenpropit in the presence of 10 μM forskolin. Data points are mean ± s.e.m. of triplicate values from single experiments that are representative of a) 5, b) 5, c) 4, d) 5 and e) 4 separate experiments.

The ligands were then examined in the ^3^H-cAMP accumulation assay and again, the agonist actions of the ligands was clearly observed (Table 1). The antagonist affinity measurements of two of the antagonists, clobenpropit and thioperamide, were then examined in the ^3^H-cAMP accumulation assay (Figure 5, Table 4). Given that VUF 5681 and burimamide were more potent (i.e. left-shifted) in the ^3^H-cAMP accumulation assay, pK_D _values were also able to be determined when these ligands were the agonists (Table 4).

**Table 4:** pK_D _values for clobenproprit and thioperamide

	pK_D_		pK_D_	
agonist	clobenpropit	n	thioperamide	n
				
histamine	9.38 ± 0.12	5	7.38 ± 0.04	4
N-α-methylhistamine	9.43 ± 0.14	5	7.28 ± 0.06	3
R-α-methylhistamine	9.36 ± 0.08	4	7.42 ± 0.09	3
S-α-methylhistamine	9.46 ± 0.08	4	7.45 ± 0.09	3
imetit	9.28 ± 0.07	5	7.48 ± 0.13	4
immepip	9.34 ± 0.15	5	7.46 ± 0.14	4
immethridine	9.32 ± 0.14	5	7.46 ± 0.13	4
proxyfan	9.32 ± 0.03	5	7.33 ± 0.09	4
VUF 8430	9.42 ± 0.10	5	7.43 ± 0.09	4
impentamine	9.46 ± 0.11	4	7.54 ± 0.09	4
burimamide	9.25 ± 0.14	4	7.39 ± 0.12	4
VUF 5681	9.20 ± 0.09	4	7.45 ± 0.10	4

pK_D _values for clobenpropit and thioperamide as determined from ^3^H-cAMP accumulation measurements made in the presence of the 12 different agonists. Values represent mean ± s.e.mean of n separate experiments.

### Impentamine

**Figure 6:**
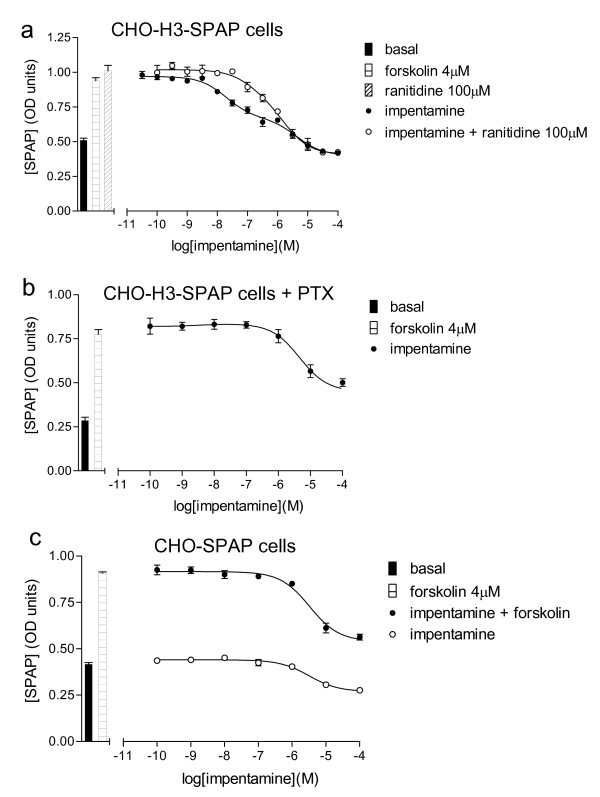
**a) CRE-SPAP production in forskolin-stimulated CHO-H3-SPAP cells in response to impentamine in the absence and presence of 100 μM ranitidine.** Bars show basal CRE-SPAP production, that in response to 4 μM forskolin alone and to 100 μM ranitidine in the presence of 4 μM forskolin. b) CRE-SPAP production in forskolin-stimulated CHO-H3-SPAP cells in response to impentamine following 24 hours pre-incubation with PTX. Bars show basal CRE-SPAP production, that in response to 4 μM forskolin alone following pre-incubation with PTX. c) CRE-SPAP production in CHO-SPAP cells in response to impentamine either alone or in the presence of 4 μM forskolin. Bars show basal CRE-SPAP production, that in response to 4 μM forskolin alone. Data points are mean ± s.e.m. of triplicate values from a single experiment and are representative of a) 5, b) 3 and c) 3 separate experiments.

Impentamine stimulated a CRE-gene transcription response that was best described by a two-component concentration response curve pIC_50 _= 7.59 ± 0.05, n = 8 and pIC_50 _= 5.40 ± 0.05, n = 8, Figure 6a). When examined in the presence of any of the antagonists used in this study, only the first component was inhibited and this yielded a pK_D _value for each antagonist the same as those obtained in the presence of all other agonists (Table 2). When the impentamine response was examined following pre-incubation with PTX, the first component was abolished, and only the second component remained (pIC_50 _= 5.22 ± 0.22 n = 3, Figure 6b). Furthermore, this low potency component was seen in the absence of forskolin in CHO-H3-SPAP cells both with (pIC_50 _= 5.29 ± 0.17, n = 3) and without PTX pre-incubation (pIC_50 _= 5.18 ± 0.29, n = 3) and in CHO-SPAP cells (i.e. without the receptor) both with (pIC_50 _= 5.33 ± 0.08, n = 3) and without forskolin (pIC_50 _= 5.41 ± 0.06 n = 4, Figure 6c). Following 5 hours incubation with impentamine, CHO-H3-SPAP cells did not show any intracellular uptake of trypan blue. Finally, the ^3^H-cAMP accumulation response is best described by a one-component sigmoidal concentration response curve, the low potency component not being seen at all. Thus at high concentrations, impentamine appears to have been inhibiting the CRE-SPAP transcription or translation rather than appearing toxic to the cells, as they did not take up trypan blue following 5 hours incubation with impentamime and the ^3^H-cAMP accumulation response remained intact (i.e. no drop below basal that might have been expected with cell death). Taken together, this suggests that the first component of the impentamine response (Figure 6a) is due to partial agonism via the H3 receptor and the second component due to a non-specific non-receptor mediated inhibition of the CRE-SPAP response downstream from cAMP, and therefore unlike the two-component responses seen at either the β1-adrenoceptor (i.e. Figure 7 of [12]) or the β3-adrenoceptor (Figure 7 of [19]).

**Figure 7:**
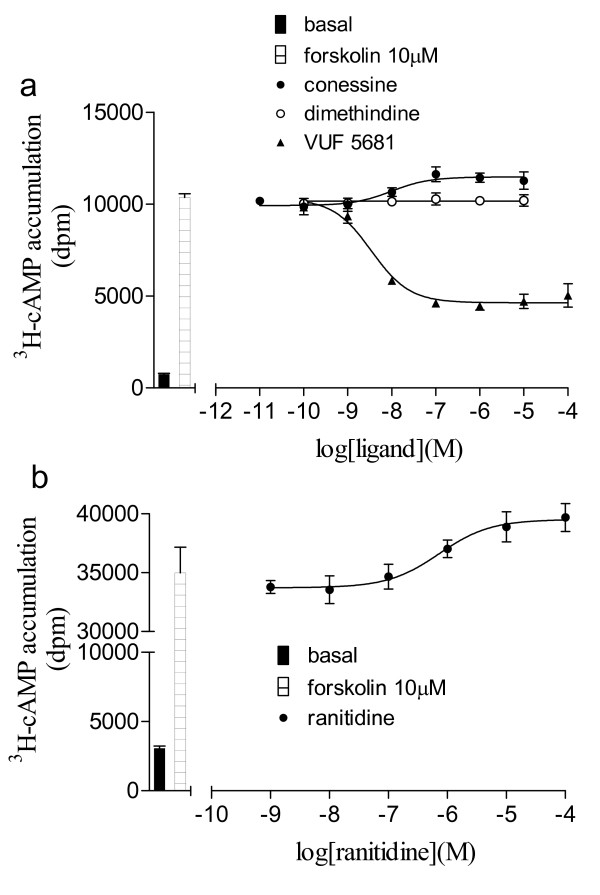
**Forskolin-stimulated ^3^H-cAMP accumulation in CHO-H3-SPAP cells in response to a) conessine, dimethindine and VUF 5681 and b) ranitidine following 5 hours ligand stimulation.** Bars show basal ^3^H-cAMP accumulation and that in response to 10 μM forskolin alone. Data points are mean ± s.e.m. of triplicate values from single experiments that is representative of 4 separate experiments in each case.

### Investigation of the intrinsic activity of the H3-antagonists

Finally, as the histamine H3 receptor is known have constitutive activity (e.g. [38], the intrinsic efficacy of the antagonist ligands was examined. With the exception of the VUF 5681 (Table 1), none of the 11 other antagonist ligands stimulated a change in CRE-SPAP production in CHO-H3-SPAP cells. However, previous studies have however demonstrated that small changes may be more clearly seen in the ^3^H-cAMP accumulation assay due to the larger response window. All antagonist ligands were therefore examined in the ^3^Hc-AMP accumulation assay following 5 hours incubation with ligands in order to maximise any changes seen. In this assay, the IC_50 _values for the partial agonist VUF 5681 remained unchanged (pIC_50 _= 8.42 ± 0.05, n = 3). Inverse agonist responses were seen for conessine (pEC_50 _= 8.51 ± 0.20, 120 ± 1.5% basal, n = 4), thioperamide (pEC_50 _= 7.53 ± 0.11, 106.3 ± 2.6% basal, n = 4), ranitidine (pEC_50 _= 5.98 ± 0.21, 112.5 ± 2.0% basal, n = 4) and cimetidine (pEC_50 _= 6.62 ± 0.24, 110.7 ± 3.0% basal, n = 4; Figure 7). No responses were seen to zolatidine, dimethindine, iodophenpropit, clobenpropit, triprolidine, clemastine or chlorpheniramine (n = 4 for each ligand).

### Lack of gene transcription responses in CHO-SPAP cells

The response to all ligand used in this study was examined in the parent CHO-SPAP cells (expressing the reporter but not the human H3 receptor). Full 7-point concentration response curves were constructed, in the presence and absence of 4 μM forskolin. No responses were seen in response to any ligand (except impentamine mentioned above), up to concentrations of 100 μM (or 10 μM in the case of zolatidine, conessine, iodophenpropit, clobenpropit, clemastine). A small decrease from maximum was seen only at the highest concentration (100 μM) of VUF 5681.

## Discussion

Several GPCRs have now been shown to exist in several different conformations, stablised by different agonists, that either couple to different G-proteins, signal via different pathways or to which ligands bind with different affinities [39]. The histamine H3 receptor is a Gi-coupled receptor with a large range of ligands of varying efficacies thus making a detailed pharmacological study of the receptor possible. Several newer ligands have also been found for the H3 receptor and their structure is becoming increasingly diverse (e.g. conessine, a steroidal alkaloid from the stem bark of Funtumia elastica that has both antimicrobial activity against Plasmodium falciparim and H3 antagonist properties [40,41]. The histamine H3 receptor has also been proven to exist in different agonist states or conformations. It is a constitutively active receptor and thus can exist in an inverse agonist state. In addition, proxyfan has been demonstrated to be a protean agonist [33]. It stabilises a medium-efficacy state of the receptor which is distinct from the high efficacy histamine induced state [33]. The purpose of this study was therefore to examine the pharmacology of the H3 receptor, and in particular antagonist affinity measurements made in the presence of agonists of different efficacies, including that state induced by proxyfan.

From the range of ligands investigated, many H3 agonists were identified. As well as the well-known ligands, others e.g. burimamide and impentamine were found to be agonists in keeping with a previous study in recombinant cells [38]. In this study, in keeping with the previous study in recombinant cells [33], proxyfan was found to be a partial agonist when formation of cAMP was examined as well as down-stream CRE-gene transcription. VUF 8430 was reported to be a low potency full agonist at the H3 receptor [47] and was also found to be so in this study. In addition, and in contrast to other studies (e.g. [47]), other ligands were also found to have agonist properties e.g. the H2 agonists amthamine and dimaprit. VUF 5681 was previously reported to be a neutral antagonist of the histamine H3 receptor [43-45]. However, here it was found to have agonist activity in both the ^3^H-cAMP accumulation assay and the CRE-SPAP assay. As all of these responses did not occur in CRE-SPAP cells (without the H3 receptor) and their responses were sensitive to pre-incubation with pertussis toxin (PTX), they too were occurring via the histamine H3 receptor.

In view of the fact that the human histamine H3 receptor is known to be constitutively active [38], evidence for this was sought in terms of demonstrating inverse agonist action of the antagonist ligands. Given the low expression level of this cell line very little constitutive activity would be expected to be seen, and indeed this was the case. Only conessine, thioperamide, ranitidine and cimetidine were found to be inverse agonists in this relatively low expressing cell system. However, this nonetheless confirmed the constitute nature of this receptor. As there was relatively little constitutive activity, this should mean that a protean agonist would appear as a more efficacious agonist, as indeed proxyfan did. Because of this, antagonist affinity measurements from parallel shifts of an agonist response were more easily determined. It is interesting to note that several histamine ligands were found to have significant agonist activity in this cell system that has not been previously reported e.g. VUF 5681. It could therefore be that VUF 5681 is also a protean agonist and if examined in systems of different receptor expression and constitutive activity (as in [33]) this might be demonstrated. Proxyfan appears as a full agonist in this cell system and VUF 5681 as a partial agonist. If VUF 5681 is indeed a protean agonist, it stabilises a different, lower efficacy protean state of the receptor to that stabilised by proxyfan.

All agonists at the H3 receptor appeared more potent in the ^3^H-cAMP accumulation assay than in the CRE-gene transcription assay. This is in contrast to a recent study of the Gs-coupled human histamine H2 receptor (where all agonist responses were more potent in the CRE-gene transcription assay; [25]), but is similar to that seen in the Gi-coupled adenosine A1 receptor [18]. Potencies between assays appear similar at the β1 and β3-adrenoceptor (except for weak partial agonists) but vary depending on the efficacy of the agonist at the β2-adrenoceptor. The reason for the change in potency between assays, that clearly varies with different GPCRs, remains unknown but may be related to different patterns of phosphorylation, internalisation and desensitisation that occur at different GPCRs [46,47].

Another major conclusion that can be drawn from this study is that not all Gi-coupled receptors behave alike in a recombinant cell system. The Gi-coupled adenosine A1-receptor, expressed in the same CHO-reporter cell back ground as used in this study, clearly showed the receptor coupling to Gi and Gs-proteins [18]. In this H3 study, all ligands were first assessed for their agonist activity in the presence and absence of forskolin, with and without PTX pre-incubation. Inhibitory responses were only ever seen. All of these responses were only seen in the presence of forskolin and all responses were abolished by pre-incubating the cells with PTX. The H3 receptor therefore did not demonstrate any Gs-coupled stimulatory CRE-gene transcription responses, even at high agonist concentrations, and thus has a different agonist receptor activation profile to the A1-adenosine receptor.

The affinity for the antagonists, as measured from parallel shifts of the agonist concentration response curve, remained constant at the human H3 receptor. Where values are available, the antagonist affinity values obtained here are similar to those previously published e.g. [26,29]. This was also true for proxyfan stimulated responses. Conessine, a steroidal alkaloid and a natural product from the stem bark of Funtumia elastica, was a high affinity H3 inverse agonist and the pK_D _values for its antagonism of different H3-agonists obtained here were very similar to that reported by Cowart et al., [41]. Thus, regardless of which competing agonist was used, the antagonist affinity of a given antagonist remained the same. Furthermore, the Schild plots, where possible, all had a slope of 1 confirming competitive antagonism. Again, where possible, this was true for the proxyfan stimulated responses. This was true even when examining the antagonism of partial agonist responses (e.g. burimamide, VUF 5681 and impentamine). This also held even when the antagonism of full agonists by a partial agonist (VUF 5681) was examined. Finally, the antagonist affinity values obtained were the same in the ^3^H-cAMP accumulation assay as the CRE-gene transcription assay. Therefore, the histamine H3 receptor is unlike any of the β-adrenoceptors in terms of simple antagonist affinity measurements. Antagonist affinity measurements remain constant at the H3 receptor, regardless of the competing agonist's efficacy, the cellular response measured, the time of incubation of agonist or the presence of a PDE inhibitor.

## Conclusion

In conclusion, the human histamine H3 receptor is a Gi-coupled receptor that, in contrast to the human A1-receptor, has no evidence of coupling to Gs-proteins in this low receptor expressing recombinant cell system. Several ligands were identified as having agonist activity, including some ligands previously considered to be antagonists (e.g. VUF 5681, dimaprit). Given the low constitutive activity of this cell system, these ligands might yet turn out to be protean agonists. Conessine was shown to be a high affinity inverse agonist. Finally, the competitive nature of the antagonists was demonstrated and antagonist affinity measurements were constant for each antagonist, including at the proxyfan-induced medium-efficacy state of the H3 receptor, in contrast to all three subtypes of the β-adrenoceptors, but in keeping with the traditional pharmacological dogma.

## Abbreviations

cAMP, adenosine-3',5'-cyclic monophosphate; CHO, Chinese hamster ovary; CRE, cyclic AMP response element; DMEM/F12, Dulbecco's modified Eagles medium/nutrient mix F12; GPCR, G-protein coupled receptor; HTMT, histamine trifluoromethyl toluidide; Nαmh, Nα-methylhistamine; PBS, phosphate buffered saline; PEA, 2-pyridylethylamine; pNPP 4-nitrophenyl phosphate; PTX, pertussis toxin; Rαmh, R-α-methylhistamine; Sαmh, S-α-methylhistamine; SPAP, secreted placental alkaline phosphatise; VUF 5681, 4-[3-(1*H*-imidazol-4-yl)propyl]piperidine dihydrobromide; VUF 8430, 2-[(aminoiminmethyl)amino]ethyl carbamimidothioic acid ester dihydrobromide.

## Authors' contributions

JGB conceived the study, carried out the experiments, data analysis and preparation of the manuscript.
